# Sanitary Convention at Austin

**Published:** 1906-05

**Authors:** 


### Sanitary Convention at Austin.

Pursuant to call of State Health Officer Geo. R. Tabor, a large number of city and county health officers of Texas assembled in Austin May 2d, representing nearly every section of the State. The editor of the TEXAS MEDICAL JOURNAL was out of the city and could not attend, hence we take this report from the secretary's minutes and the Galveston *News:*

The meeting was called to order by Dr. Tabor, who stated that the object of the meeting was to discuss sanitary and quarantine matters with the view of benefiting the public. He complimented the local health officers for the assistance they had rendered him in the campaign against yellow fever. The Governor granted every request made of him for aid in enforcing the quarantine, but there was a lack of legislative appropriation for the health department. He said that his department is extremely anxious over the possibility of a recurrence of yellow fever in Louisiana this year owing to the mildness of the last winter. Texas was also menaced by Havana, and he has every reason to believe, he said, that yellow fever exists in that city at this time. There is an epidemic of dengue fever there now and some of the cases have proved fatal. The Louisiana authorities claim that yellow fever was introduced into that State last year through the carelessness of the Federal government.

Dr. Trueheart, the veteran city health officer of Galveston and yellow fever expert, read a paper (contributed to the TEXAS MEDICAL JOURNAL, and published herewith) on the subject of the importance of exterminating the yellow fever mosquito. He prefaced his remarks by saying that he recently spent three weeks in San Antonio, where he made a diligent search for the yellow fever mosquito. He was only able to find three of this breed of mosquitoes in that city. He told the health board of San Antonio that he had found the sanitary condition of that city so perfect that he had no suggestions to offer for further improvement. The paper which Dr. Trueheart read was an able treatise on the yellow fever mosquito and the methods for its extermination. He was opposed to the plan of secrecy which has been observed in some cities when yellow fever appeared. He urged that the public be advised of all cases of the disease with the view of obtaining their co-operation in exterminating the disease. There should be no necessity of quarantine being established if sanitary regulations are adopted and enforced in every community, such as are found at San Antonio and Galveston.

Dr. Tabor discussed the paper, briefly pointing out the importance of exterminating the yellow fever mosquito.

Dr. Trueheart replied to many questions as to the method of disinfecting buildings, closets, etc.

Dr. Charles Galloway, of North Fort Worth, said he had the hardest proposition of any health officer in the State. He said that he could not enforce sanitation regulations and did not meet with support in an effort to better conditions.

"The packers say that if you push things on them they will leave the State," he continued. "Of course, that is only a bluff. The committee on health advise and I condemn. We have a city attorney who I am afraid has not enough backbone to give much help. If I can't handle the packers in the matter of making them quit dumping their refuse matter within a few blocks of the business district of North Fort Worth, I will turn them over to the county health officer, and if he doesn't do anything, I will turn them over to Dr. Tabor. I think the best thing that could happen to North Fort Worth would be an epidemic of yellow fever; it would teach the people there a lesson in sanitation matters."

Dr. Tabor assured Dr. Galloway that he would be glad to confer with him on the subject.

Dr. C. H. Irion, president of the State Health Board of Louisiana, made a talk on the situation in Louisiana as applied to what has been done to prevent a recurrence of yellow fever in that State. He said that there is no disposition on the part of the physicians to keep secret yellow fever cases, although this might have been the practice in the past. Although there was yellow fever in New Orleans as early as June 10, the first official announcement that it existed there was not made until July 21 last year. Many of the most prominent physicians were absolutely unaware of the presence of yellow fever in New Orleans on July 13. Dr. Irion gave a review of the methods employed to keep down a recurrence of the epidemic.

When the session of the County and City Health Officers' Assembly convened after the noon recess, Dr. Tabor called on Dr. H. D. Barnitz, president of the San Antonio Board of Health, for an outline of sanitary conditions in that city.

### SANITATION IN SAN ANTONIO.

Dr. Barnitz said that prior to 1897 the sanitary condition of San Antonio was bad. Since the epidemic of dengue fever in that year, however, sanitation has been a matter of first importance. He stated that the San Antonio river is not a mosquito breeding stream, as it has springs for its source and is without stagnant pools. The stream has had the attention of the board of health for quite a while now, and there is little danger in that direction.

"The board of health," said the doctor, "profited much last year by the hitch in administration officers. When the new administration came in many of the employes of the old regime continued in the service. We had two sets of firemen and police. These the health department utilized during the yellow fever scare."

The doctor expatiated on the system of the warfare against disease in San Antonio. The eight inspectors are ever alert and will trace down and exterminate any unsanitary condition likely to produce disease-breeding germs. Dr. Barnitz said that San Antonio is one of the most sanitary cities in the State, and that the people have at last been educated up to the point where they are of great assistance to the board of health, and invariably report any breach of the sanitary rules that comes under their observation.

Dr. S. Burg, city health officer of San Antonio, went more into detail as regards the situation in that place than did his colleague.

"Since 1903," said Dr. Burg, "we have ever waged an aggressive campaign against disease. Circulars were posted telling the populace how to maintain sanitary premises, and advising them what to do and what not to do. We had eighteen or twenty fumigating wagons in operation constantly during the period of apprehension. We succeeded in abolishing 300 to 400 cisterns throughout the city. A thorough system of sewer cleansing is successfully operated with the assistance of the fire department. The sewers are flushed and the manholes oiled. We now have a pure system of sewerage. The city authorities are with us, and have assisted the good work beyond measure. In fact, the secret of perfect sanitation is the harmonius co-operation of the mayor and the council."

Dr. Tabor, in commenting upon Dr. Burg's remarks, declared that he was perfectly familiar with the effectiveness of the campaign in San Antonio against the mosquito.

SAN ANTONIO AND GALVESTON.

"I don't believe," said Dr. Tabor, "that a yellow fever epidemic could thrive in San Antonio or Galveston."

Dr. Tabor took the opportunity to reply to Dr. Galloway's complaint against the council of Fort Worth.

"There is only one way to master the situation," said the doctor. "File your complaint and get the city attorney to prosecute the case to the end. Get public sentiment with you. Let it be understood that no one, however high politically or financially he may be, can violate the sanitary laws and go unpunished." (Applause.)

Dr. Bryan, of Hillsboro, spoke briefly on sanitary conditions in his town.

Dr. Braswell, of Mineral Wells, during the progress of his remarks, stated that he believed the time was near when quarantine will be a thing of the past.

"Wage your fight against the mosquito, and if possible quarantine it, and you have the secret of preventing the spread of yellow fever," said Dr. Braswell.

Dr. Thomas Moody, county health officer of Lamar county, concurred in Dr. Braswell's theory that the efforts of the health department should be primarily directed against the stegomyia. He also stated that he could not get a jury to punish violators of the sanitary laws, as they invariably ask if any spread of disease resulted from such violation, which, of course, can rarely be proved.

Dr. R. C. Hall, city health officer of Marshall, spoke at length on conditions in that city.

OPPOSE FEDERAL CONTROL.

At this juncture Dr. Moody brought up the matter of Federal control of quarantine, saying that while he was opposed to the proposition, there were many in his community who had sought to make him promise to urge his influence for such a change at this meeting.

Dr. Tabor stated that he believed the Federal government had no constitutional right to take the prerogative of quarantine policing away from a State.

"I am for the people always," said the doctor, his remarks being greeted by applause.

He then went on to show that the people of Texas opposed Federal control by reason of the fact that both Houses of the Legislature had passed concurrent resolutions at the last special session congratulating the Texas representation in Congress in their opposition to such control.

"Such action by the Legislature," said Dr. Tabor, "is a pretty fair illustration of the sentiment of the people."

Dr. Burg, of San Antonio, declared himself in favor of home control, but thought the State should make appropriations sufficient to meet the necessary demands of the quarantine department for an efficient and effective operation of its system.

Dr. Joseph Gilbert, city physician of Austin, then offered a motion that the body commend the action of the Texas delegation in Congress in their fight against Federal control, which was unanimously adopted by a rising vote.

LACK OF APPROPRIATIONS.

In speaking of the lack of appropriation for the successful operation of the laws pertaining to the health department, Dr. Tabor stated that an accumulation of two years' standing of vital statistics was stored in his office, undisposed of for want of funds.

"I could not get a $75 clerk to take charge of these records," said Dr. Tabor.

The doctor stated further that the pure food law provided for the employment of an expert chemist by the health department for the purpose of carrying on analyses, but not a cent was appropriated with which to pay him.

On motion of Dr. Braswell the chair was authorized to appoint a committee of five to urge further appropriations for the health department upon the next Legislature.

Drs. LeMaster, of Goliad, Higgins, of Greenville, and others made statements as to the sanitary conditions of their respective localities.

The question of making charges for health certificates was brought up. Dr. Higgins and Dr. West, of Fort Worth, stated that they charged for health certificates which they issued.

Dr. Tabor said that he had repeatedly said that he is opposed to graft in health certificates; that he had said nothing to apologize for. He said that he did not believe the work should be done free, but the graft he referred to was the issuing of certificates by physicians to persons they had never laid eyes upon before. He took the position that such certificates should be issued to persons who are known; that the cities and towns should employ a clerk for the purpose of issuing them.

Dr. West interrupted to say that Dr. Tabor gave an interview in which he used some severe and harsh language, which he felt reflected upon him personally. This interview was published last year during the yellow fever epidemic in New Orleans. Dr. Tabor said that he said nothing in that interview that he had not repeated upon the floor at this meeting.

Dr. Higgins said that he charged $1 straight for the certificates which he issued, and that he did not think Dr. Tabor's interview applied to him.

After further discussion the meeting adjourned.

Those who were present are: S. M. Stephens, Beeville; J. J. Adkins, Refugio; G. M. Braim, Hillsboro; R. O. Braswell, Mineral Wells; Thomas Moody, Paris; R. C. Hall, Marshall; R. R. Le Master, Goliad; D. M. Higgins, Gainesville; I. G. Langston, Waco; A. W. W. Cox, Laredo; P. D. Barnhill, Brenham; G. L. Wilie, West; J. E. Baldwin, Dallas; S. Burg, San Antonio; G. W. Allen, Jr., Yorktown; A. J. Wynatt, Liberty; T. R. Fisher, Dallas; Stephen T. Beasley, Crockett; H. D. Barnitz, San Antonio; C. W. Trueheart, Galveston; T. W. Largent, Lufkin; R. B. West, Fort Worth; F. J. Gilson, Calvert; William Erwin, Hearne; W. F. McMullen, Rockport; C. H. Irion, New Orleans; W. R. P. Thompson, Sabine; Joe Gilbert, Austin; H. P. Lockett, Bastrop; J. G. Pope, Robinson, Coleman county; Charles Galloway, North Fort Worth.

